# Unusual presentation of oral hemangioma in tongue and the potential use of propolis as an adjunctive treatment

**DOI:** 10.1002/ccr3.5243

**Published:** 2021-12-22

**Authors:** Susan Susan, Munir Ravalia, Felix Zulhendri

**Affiliations:** ^1^ Private Practice Medan Indonesia; ^2^ The Royal London Hospital Whitechapel, London UK; ^3^ Scientific Consultant North Sumatra Indonesia; ^4^ Center of Excellence in Higher Education for Pharmaceutical Care Innovation Universitas Padjadjaran Jatinangor Indonesia; ^5^ Physiology Division Department of Biomedical Sciences Faculty of Medicine Universitas Padjadjaran Bandung Indonesia

**Keywords:** analgesic, anti‐inflammatory, benign tumor, haemangiomas, propolis, vascular, wound healing

## Abstract

Tongue hemangioma is a rare case of vascular tumors that causes symptoms such as pain, bleeding, difficulty in chewing, speaking, and breathing. We report a case of an oral lobular capillary hemangioma on the dorsal surface of the tongue treated with surgical excision and postoperative application of propolis extract.

## Graphical abstract

The difficulty in diagnosing oral lobular capillary hemangiomas may potentially arise due to the confusion with other similar conditions. Histopathological assessment is still the most accurate and satisfactory means of diagnosis.

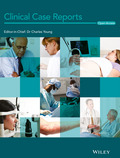

## 1 INTRODUCTION

Hemangiomas by definition are a benign tumor of blood vessels. They commonly occur in the head and neck region. In the oral cavity, hemangiomas can cause several symptoms such as pain and bleeding, and in more serious cases, can negatively impact the quality of life by hampering the ability to chew, speak, and breathe. Hemangiomas are histologically categorized into three types, namely capillary, cavernous, or mixed, where about 80% occur as single lesions and 20% as bilateral lesions. The occurrence in male‐to‐female patient ratio is approximately 1–3. They are mostly found on the cheeks, upper lip, and eyelids, and rarely in the oral cavity, especially on the tongue.^1^, ^2^, ^3^

Propolis is a beehive‐derived natural product that has been shown to have anti‐inflammatory, anti‐nociceptive, and wound‐healing properties.^4^, ^5^ Magro‐Filho and de Carvalho^6^ investigated the effect of propolis on dental sockets and skin wounds in rats and found that propolis promoted epithelial repair. In addition, they investigated the effect of propolis‐containing mouthwash on patients who underwent intrabuccal surgeries. Propolis significantly induced healing of the surgical wounds.^7^ More importantly, a systematic review by Oryan et al.^8^demonstrated the therapeutic benefits of topical application of propolis as an adjunctive therapy for wound care.

The present case report describes a case of a female patient who had a growth on her left dorsal surface of the tongue which was subsequently diagnosed as an oral lobular capillary hemangioma based on the International Society for the Study of Vascular Anomalies (ISSVA) Classification of Vascular Anomalies.^9^ She was treated with a surgical excision and postoperative application of hydroglyceric propolis extract and chlorhexidine mouthwash.

## 2 CASE PRESENTATION

**FIGURE 1:**
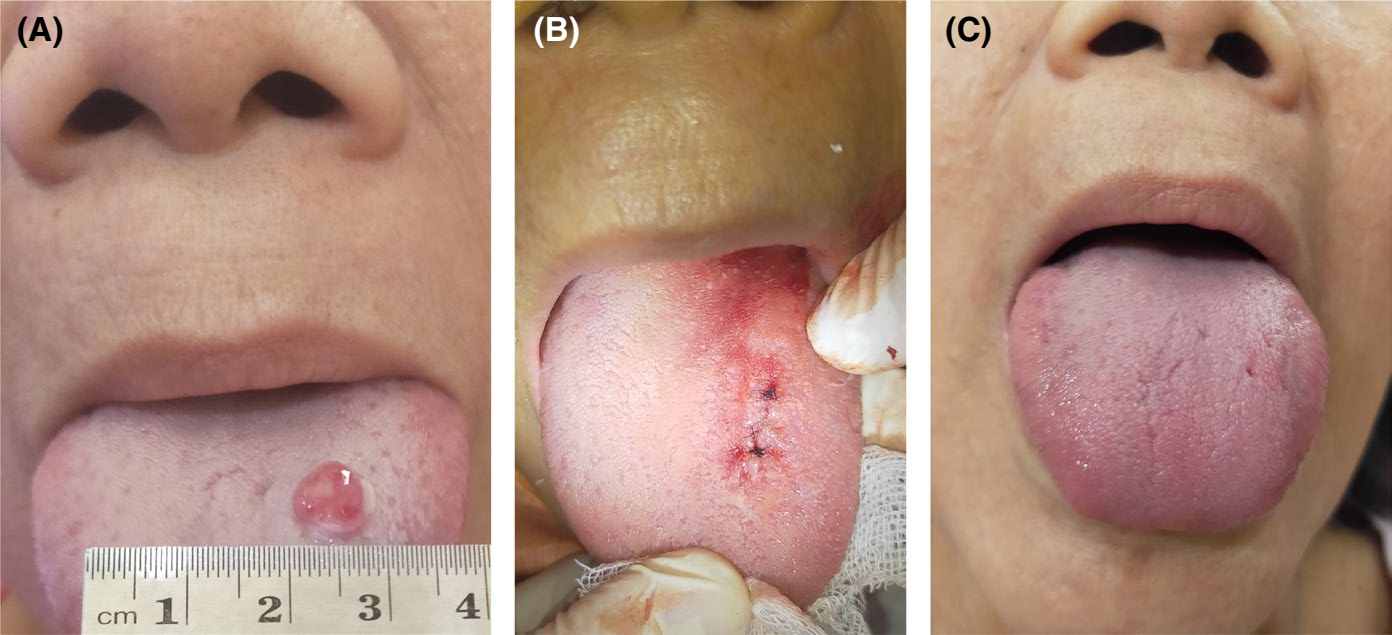
(A) Appearance of the oral hemangioma lesion. (B). Surgical removal of the lesion. (C). Complete resolution was noted after 2 weeks

A 63‐year‐old female patient in Medan, Indonesia, came to a dental clinic with a primary complaint of a lesion on the left dorsal surface of the tongue, which caused the difficulty in chewing and speaking. The lesion was of the size of a pea when the patient first noticed it but had grown to attain the size of 8 × 7 × 3 mm over a period of 2 weeks. The lesion had started to bleed when she was presented to the clinic. The patient's medical history was unremarkable. She mentioned that she previously went to a general practitioner and was prescribed amoxicillin, dexamethasone, and mefenamic acid. She consumed the medications for 5 days and reported no improvement. Intraoral examination revealed a single, spherical‐shaped, and reddish pink lesion. The lesion had a distinct border and irregular surface (Figure 1A). The surrounding tongue mucosa appeared to be normal, and the growth was located on the left dorsal surface of the tongue. The lesion had a soft‐to‐firm consistency and blanched upon pressure.

The management of the lesion was a surgical excision, which was carried out under local anesthesia. A thread was tied around the base of the lesion. The lesion was then stretched upward to facilitate accessibility. It was subsequently excised out, and interrupted sutures were placed (Figure 1B). During the surgical procedure, minimal amount of bleeding from the site was observed. The specimen was then sent for a histopathological examination. After the surgery, the patient was instructed to keep the oral cavity clean by teeth and tongue brushing three times a day followed by chlorhexidine mouthwash. The patient was then asked about whether she had prior history of allergic reactions in relation to the use or consumption of beehive products such as honey, bee pollen, or propolis. She confirmed that she never had any allergic reaction to any beehive product. The patient was then instructed to also apply the commercially available hydroglyceric propolis extract to the surgical wound three times a day for a week. In addition, the patient was prescribed 500 mg mefenamic acid and instructed to consume when only the postoperative pain became unbearable. The partial healing was observed after a period of 1 week, and complete healing was observed a fortnight after the surgical procedure (Figure 1C). In our experience, the postoperative wound‐healing period for this type of surgical intervention would usually be, on average, at least twice as long.

**FIGURE 2:**
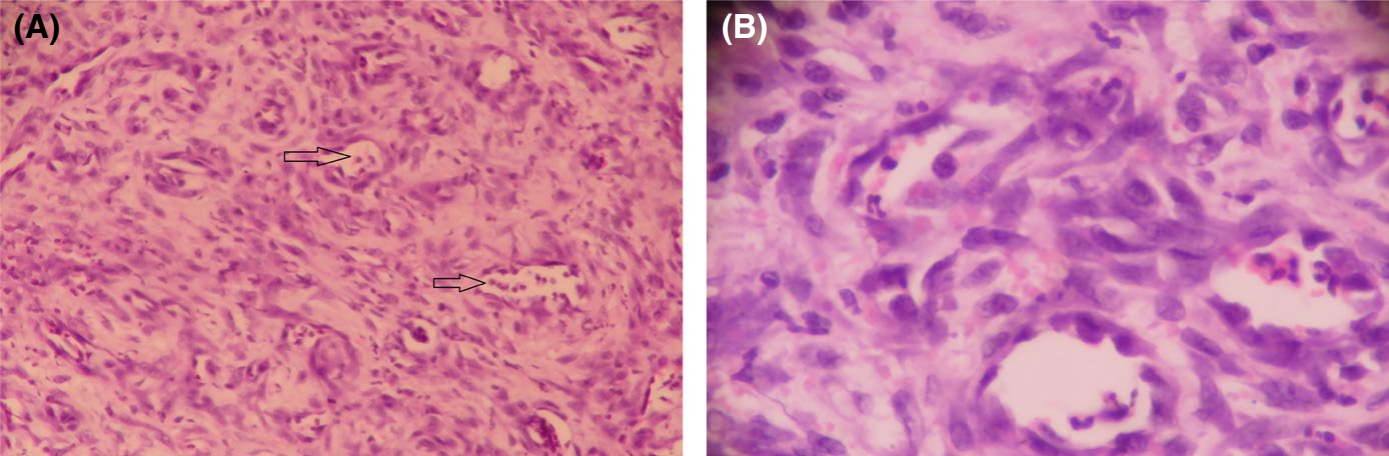
(A) Leukocyte infiltrates in the blood vessel lumen (Hematoxylin and eosin staining, 100×). (B) Erythrocyte and leukocyte infiltrates in blood vessel lumen (Hematoxylin and eosin staining, 400×)

The histopathological section of the specimen showed the characteristics of an ill‐defined intramuscular nodule with a pattern that was consistent with increased vascularity and inflammation. It was dominated by the appearance of epithelioid cells where the nuclei were in normal form. The predominant sub‐epithelium consisted of tubular, proliferating blood vessels where the lumen was partially filled with red blood cells. The stroma was composed of fibrous connective tissue within the normal range which had a moderate amount of inflammatory white blood cells infiltrate. No signs of malignancy were found in the specimen. The histopathological diagnosis was given as lobular capillary hemangioma by a qualified pathologist (Figure 2A,B).

## 3 DISCUSSION

The difficulty in diagnosing oral hemangiomas may potentially arise due to confusion with other similar conditions, such as other benign, pre‐malignant, and malignant tumors. Additionally, vascular anomalies of head and neck have historically confused clinicians due to the inconsistent nomenclature.^2^, ^10^ Consequently, improper diagnoses and inappropriate treatments may be applied to the detriment of the patients. In the present case, a misdiagnosis by a general practitioner occurred. The oral lobular capillary hemangioma was improperly diagnosed as a microbial infection and/or inflammation and judged by the prescription given to the patient. Histopathological assessment is still the most accurate and satisfactory means of diagnosis. Additional radiographic analyses may be advised to rule out any malignancy, any bony destruction related to the central variety of hemangiomas, and to identify any foreign body that may be required to be removed with the lesion.^10^ Moreover, the ISSVA classification is an invaluable and excellent tool that should be utilized whenever vascular anomalies are encountered. In addition to standardizing the classification of vascular anomalies, the ISSVA classification is also a useful tool in differential diagnosis.^9^

The oral lobular capillary hemangioma lesion in the present case appeared to be relatively benign and non‐life threatening. However, the patient complained about bleeding and having difficulties in chewing and speaking. Therefore, simple excision was ordered under necessary precautions. Where possible, laser and cold surgeries can also be considered as these surgical methods have been shown to be effective and result in less postoperative complications such as less bleeding.^11^, ^12^, ^13^

Propolis was considered as an adjunctive treatment in the present case because of its antimicrobial, anti‐inflammatory, anti‐nociceptive, and wound‐healing properties. The application of propolis was well‐suited to the present case. Propolis has been demonstrated to be useful in treating many oral diseases and disorders in various clinical trials. Several blinded, randomized, placebo‐controlled studies demonstrated the efficacy of propolis in preventing and reducing the severity of oral mucositis.^14^, ^15^

More importantly, propolis has also been shown to promote faster surgical wound healing in oral cavities. Lisbona‐Gonzales et al.^16^ demonstrated that 90% of the enrolled patients who were treated with propolis toothpaste in combination with chlorhexidine mouthwash following teeth extraction in periodontal disease showed complete healing after 3 days, whereas only 13% of the control patients (treated with only chlorhexidine mouthwash) had complete healing. In addition, Moon et al.^17^ showed that propolis significantly reduced the incidence of post‐tonsillectomy hemorrhage and postoperative pain. The wound healing was also significantly better in the propolis‐treated patients. Furthermore, a systematic review of clinical trials by Oryan et al.^8^ appeared to support the use of propolis in wound care. However, propolis is a known allergen and contact allergy, allergic contact dermatitis, and allergic contact stomatitis, albeit rare, have been shown to be linked to propolis use and consumption.^18^, ^19^

In the present case, the patient was ordered to apply the commercially available hydroglyceric extract of Indonesian stingless bee propolis on the surgical wound three times a day. She noted the lack of pain in the surgical wound to the point that she opted to not consume the prescribed mefenamic acid. She also had a noticeable improvement in terms of wound healing within a week after the surgery. Propolis appears to be a suitable adjunctive treatment for oral application in the postoperative recovery. The present case was followed up for 6 months after the surgery. The wound healing was complete, and no recurrence was noted. Caution must always be applied in utilizing any bee product as an adjunctive therapy as allergic reactions might occur.

## 4 CONCLUSIONS

Capillary hemangiomas are infrequently seen on the tongue mucosa and can easily be confused with other lesions such as malignant tumors. Therefore, appropriate diagnoses and management by oral maxillofacial and dental surgeons are critical. Histopathological assessment remains the most accurate and satisfactory means to resolve ambiguity. In addition, propolis has been shown to have potent antimicrobial, anti‐inflammatory, analgesic, and wound‐healing properties. The present case demonstrates the potential use of propolis as an adjunctive treatment for postoperative application in the oral cavity.

## CONFLICT OF INTEREST

All other authors declare no competing financial interests and no conflict of interest.

## AUTHOR CONTRIBUTIONS

S.S. and F.Z. investigated the study and wrote original draft. M.R. involved in review and editing. All authors approved the final manuscript.

## CONSENT

Written patient consent has been signed and obtained from the patient.

## Data Availability

Data sharing is not applicable to this article as no datasets were generated or analyzed during the current study.
