# Correction to “Unusual Presentation of Oral Hemangioma in Tongue and the Potential Use of Propolis as an Adjunctive Treatment”

**DOI:** 10.1002/ccr3.72014

**Published:** 2026-02-10

S. Susan, M. Ravalia, and F. Zulhendri, “Unusual Presentation of Oral Hemangioma in Tongue and the Potential Use of Propolis as an Adjunctive Treatment,” *Clinical Case Reports* 9, no. 12 (2021): e05243, https://doi.org/10.1002/ccr3.5243.

Correction:

The authors would like to apologize for an oversight in the original publication. The conflict of interest statement was inadvertently omitted. The correct disclosure is provided below:

Felix Zulhendri is affiliated with Kebun Efi, a company that produces Indonesian stingless bee propolis. Kebun Efi did not fund the study and had no involvement in the study design, data collection, analysis, interpretation, or manuscript preparation.

We sincerely apologize for this omission and any confusion it may have caused.

